# Hypoxic Therapy as a New Therapeutic Modality for Cardiovascular Benefit: A Mini Review

**DOI:** 10.31083/j.rcm2305161

**Published:** 2022-04-28

**Authors:** Hun-Young Park, Sung-Woo Kim, Won-Sang Jung, Jisu Kim, Kiwon Lim

**Affiliations:** ^1^Department of Sports Medicine and Science, Graduate School, Konkuk University, 05029 Seoul, Republic of Korea; ^2^Physical Activity and Performance Institute (PAPI), Konkuk University, 05029 Seoul, Republic of Korea; ^3^Department of Physical Education, Konkuk University, 05029 Seoul, Republic of Korea

**Keywords:** cardiovascular diseases, cardiovascular health, hypoxic therapy, hypoxic exposure, hypoxic training

## Abstract

Cardiovascular diseases (CVDs) are recognized as one of the major causes of morbidity and mortality worldwide. Generally, most CVDs can be prevented by addressing behavioral risk factors, including smoking, unhealthy diet and obesity, lack of physical activity, and alcohol abuse. Therefore, it is important to have a healthy lifestyle by performing regular physical activity to improve cardiovascular health and diseases. However, a majority of adults worldwide do not meet the minimum recommendations for regular aerobic exercise, and overweight and obesity ratio continues to rise. In addition, obese individuals, with a high prevalence of CVDs, have a lower participation rate for exercise because of the strain on the musculoskeletal system. Hypoxic therapy, including exposure or exercise intervention under hypoxia, has been utilized as a new therapeutic modality for cardiovascular benefit and amelioration of CVDs. Hypoxic therapy shows various physiological and pathophysiological properties, including increased appetite suppression and dietary intake reduction, increased energy consumption, improved glycogen storage, enhanced fatty acid oxidation, improved myocardial angiogenesis or ventricular remodeling, augmentation of blood flow within the skeletal muscle vascular beds, and reduction of the burden on the musculoskeletal system making it applicable to patients with CVDs and obesity with attenuated cardiovascular function. In particular, hypoxic therapy is very effective in improving cardiovascular benefits and preventing CVDs by enhancing arterial function, vascular endothelial function, and hemorheological properties. These observations indicate that hypoxic therapy may be an important and essential strategy for improving cardiovascular health and reducing cardiovascular morbidity and mortality.

## 1. Introduction 

Cardiovascular diseases (CVDs) are the leading cause of death globally [1]. An estimated 17.9 million people died from CVDs in 2019, representing 32% of all global deaths [2, 3]. Of these deaths, 85% were due to heart attack and stroke [2, 3]. Of the 17 million premature deaths (under the age of 70 years) due to noncommunicable diseases in 2019, 38% were caused by CVDs [4]. Most CVDs can be prevented by addressing behavioral risk factors such as tobacco use, unhealthy diet and presence of obesity, physical inactivity, and alcohol abuse [5]. It is important to detect CVDs as early as possible so that management with lifestyle modification and medication can be initiated [6].

Among the several behavioral modification therapies, physical activity or exercise is recognized as a representative strategy for reducing, preventing, and treating CVD risk factors [6, 7, 8, 9]. Physical inactivity is a significant risk factor for CVDs [10]. Sedentary individuals have a 150–240% higher risk and prevalence for CVD than physically active individuals [7]. The main benefit of being physically active in reducing the risk of CVDs is that the individuals who are even slightly physically active, regardless of the amount of physical activity, have lower morbidity and mortality rates than those who are not physically active [9]. These findings suggest that a relatively small increase in physical activity can lead to a remarkable reduction in CVDs [6, 9]. In addition, increasing the amount of physical activity also reduces the risk of stroke and heart failure due to a dose-dependent relationship [6]. Regular exercise is especially effective in lowering the risk of CVDs by improving body composition, cardiovascular parameters, and arterial function [11].

However, a majority of adults do not meet the minimum recommendations for regular aerobic exercise [6, 10, 12]. The proportion of overweight/obese individuals is increasing in both sexes and across all age groups [13]. In particular, obesity is linked to numerous diseases of the cardiovascular system and increases CVD prevalence and mortality independently of other cardiovascular risk factors [14, 15, 16]. Recent research results emphasize abdominal obesity, which is determined by waist circumference, as a representative cardiovascular risk parameter [14]. It is important to note that overweight individuals have difficulty performing exercise due to musculoskeletal problems, and thus, they suffer a vicious cycle of continuously increasing cardiovascular risk and disease prevalence [17, 18].

Currently, several researchers have widely used hypoxic conditions for cardiovascular benefit and amelioration of CVDs based on studies showing that high-altitude populations have lower rates of obesity and CVD than those who live at sea level [17, 19, 20]. Hypoxic therapy such as hypoxic exposure and hypoxic training is being used as a novel therapeutic modality to effectively reduce cardiovascular risk and obesity, which is highly associated with numerous CVDs [18, 21, 22, 23, 24, 25, 26]. In addition, hypoxic therapy is being used effectively to promote the health of various populations by improving various physiological and pathophysiological properties [23, 24, 25, 26]. Acute exercise under hypoxia reduces joint loading and improves functional ability by decreasing exercise load, especially in the same expenditure. Chronic exercise intervention under hypoxia also improves body composition, aerobic fitness, muscle function, cardiometabolic parameters, and arterial endothelial function [17, 25, 27, 28, 29, 30, 31, 32, 33, 34, 35, 36, 37]. Several previous studies have investigated the effects of hypoxic therapy such as exposure or exercise under high altitude and hypoxia on cardiovascular benefits and improvement of CVDs [24, 27, 28, 29, 30, 31, 32, 33, 34].

Therefore, this narrative review summarizes recent evidence and any possible benefits of hypoxic therapy under hypoxia in cardiovascular function and CVDs.

## 2. Physiological Responses to Hypoxia

Short-term and/or long-term hypoxic exposure induces various physiological and pathologic changes [17, 21, 23]. Acute exposure to hypoxia activates the sympathetic nervous system among the components of the autonomic nervous system (ANS) as a compensatory response leading to increases in heart rate and minute ventilation, which leads to an increase in cardiac output [27, 38]. Hyperventilation under hypoxia is regulated by peripheral chemoreceptors in the carotid arteries in response to reduced arterial oxygen partial pressure and is an essential process for supplying sufficient oxygen to tissues [38, 39, 40]. These ventilation and cardiovascular physiological responses to hypoxia allow the metabolic needs of tissues to be met at rest and during exercise under hypoxia. Prolonged exposure to hypoxia leads to erythropoiesis, which increases erythrocyte mass, leads to an adaptation of the respiratory response, and decreases cardiac output to a level similar to that in normoxia [17, 18, 41, 42].

In addition, acute exposure to hypoxia induces appetite loss and reduced dietary intake due to anorexia [43, 44, 45]. Several studies have reported that hypoxemia caused by hypoxia induces a decrease in appetite and those changes in appetite-regulating hormones under hypoxia have a particularly significant effect [22, 46, 47, 48, 49, 50]. Leptin and ghrelin are considered the most representative dietary regulating hormones that show positive changes under hypoxic exposure, and changes in several appetite-related hormones and adipokines such as glucagon-like peptide-1 (GLP-1), pancreatic polypeptide (PP), and peptide YY (PYY) have been demonstrated to be associated with hypoxic exposure [17, 22, 44, 45, 46, 47, 48, 49, 50]. Several previous studies have demonstrated that exposure and exercise intervention under hypoxia induces decreased appetite and energy intake by decreasing ghrelin and increasing leptin, GLP-1, PP, PYY, and norepinephrine [46, 47, 48, 51, 52, 53]. This means that appetite reduction by hypoxia is considered to have physiological and pathologic properties in showing the potential to prevent obesity and thereby reducing the morbidity and mortality rates in people with CVDs [17].

**Fig. 1.**
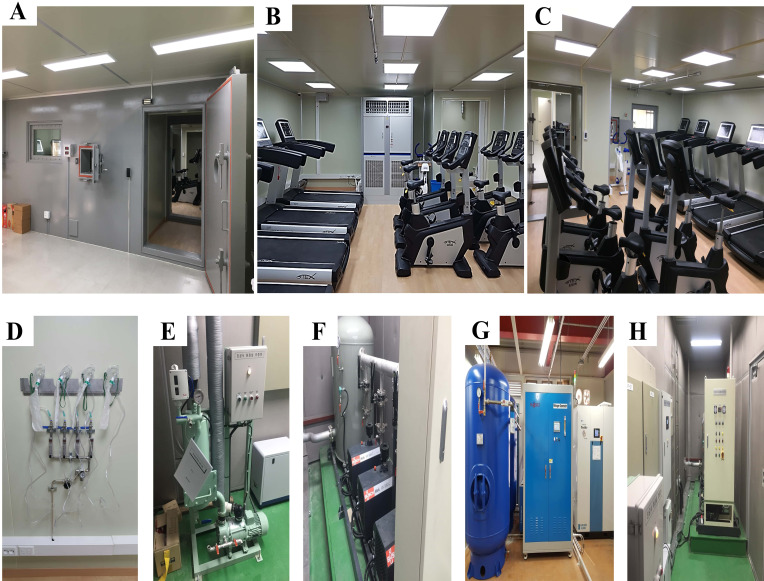
**Environmental control chamber equipment for various hypoxic therapies including exposure and exercise intervention under hypoxia**. (A) External view of the environmental control chamber. (B) Front view of the inside of the environmental control chamber. (C) Posterior view of the inside of the environmental control chamber. (D) Emergency oxygen supply equipment. (E) Vacuum toilet system. (F) Vacuum pump device for hypobaric hypoxia. (G) Nitrogen generator for normobaric hypoxia. (H) Vacuum pump panel.

The most representative methods of hypoxic therapy are exposure and/or exercise intervention under hypoxia, and they are known to induce various physiological changes [17, 21, 23, 27, 38, 39, 40, 41, 42, 43, 44, 45]. Environmental control chamber equipment for various hypoxic therapies, including exposure and exercise intervention under hypoxia, is shown in Fig. 1. Previous studies summarized the compensatory mechanisms and physiological responses to hypoxic therapy, including bodyweight responses (i.e., decreased resting leptin level, increased adrenergic response, resting norepinephrine remains post-treatment, increased serotonin level and suppressed appetite), cellular and metabolic responses (i.e., increased hypoxic inducible factor-1 and vascular endothelial growth factor expression, increased angiogenesis, increased glycolytic enzyme and number of mitochondria, improved insulin sensitivity, and increased glucose transporter-4), cardiovascular responses (i.e., increased resting and maximal heart rate, increased peripheral vasodilation, increased diameter of arterioles, increased affinity of hemoglobin to oxygen, normalized blood pressure (BP), and improved cardiovascular protection), and respiratory responses (i.e., hyperventilation, increased lung diffusion capacity for carbon monoxide and oxygen, increased carbon dioxide reserve in sleeping, decreased arterial oxygen saturation ($S_{\text{a}}$$O_{2}$), increased ventilation response during exercise, and improved respiratory function), especially hypoxic exposure [17, 20, 21, 23]. Considering cardiovascular responses among various compensatory mechanisms and physiological responses to hypoxic therapy, these might provide sufficient evidence for the cardiovascular benefit and therapeutic effect on CVDs [31, 36, 41]. In other words, hypoxic therapy, including exposure and exercise intervention under hypoxia, is considered to have the potential to reduce morbidity and mortality in people with CVDs by enhancing oxygen delivery and utilizing capacity, arterial compliance, arterial endothelial function, and hemorheological properties, thereby increasing cardiovascular benefit and improving CVD outcomes [18, 21, 23, 24, 25, 26, 27, 28, 29, 30, 31].

## 3. Hypoxic Therapy and Clinical Implications

**Fig. 2.**
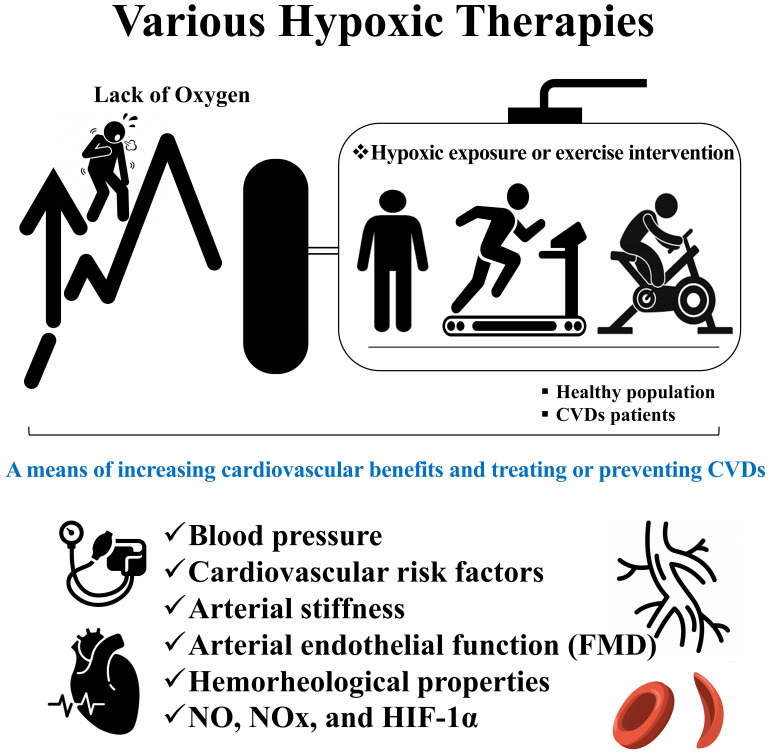
**Efficacy of hypoxic therapy in enhancing cardiovascular benefits and in the treatment and prevention of cardiovascular diseases**. CVDs, cardiovascular diseases; FMD, flow-mediated dilation; NO, nitric oxide; NOx, nitric oxide metabolites; HIF-1α, hypoxia-inducing factor-1 alpha.

One of the new therapeutic alternatives to increase cardiovascular benefits resolve CVDs is hypoxic therapy, which was recently used as a common medical modality and is positioned in the field of alternative medicine [21, 23, 24, 25]. Hypoxic therapy such as hypoxic exposure or hypoxic exercise intervention can be used to increase cardiovascular benefits and to treat or prevent CVDs (Fig. 2). Therefore, this review focuses on the effectiveness and potential applications of hypoxia therapy.

### 3.1 Hypoxic Exposure as a Therapeutic Modality for Cardiovascular Benefit

The initial data used to evaluate the relationship between hypoxic exposure and CVDs were derived from epidemiological studies. These studies compared high-altitude and low-altitude populations, and several studies showed that the risk of CVDs decreased with increasing altitude, but other studies have reported the opposite [54, 55, 56, 57, 58, 59, 60, 61]. Most of these conflicting findings result from confounding factors (i.e., ethnicity, race, sex, physical activity, and nutritional status), which were not adequately addressed in the study design and analysis. Faeh *et al*. [62] evaluated the effect of altitude on CVDs using the Swiss National Cohort Study Group data. They analyzed the relationship between altitude (e.g., hypoxia degree) and CVDs using data from 1.64 million German and Swiss residents and found a highly beneficial effect on coronary artery disease (CAD) and stroke. In addition, they reported that individuals who were born at high altitudes also had an independent protective effect for CAD [29, 62]. The beneficial effects of high altitude on CVDs have been consistently reported in other studies [63, 64, 65]. Faeh *et al*. [63] conducted a follow-up study of the Swiss National Cohort using data from 4.2 million individuals. They reported that mortality from CVDs decreased linearly with increasing altitude. Ezzati *et al*. [64] confirmed a beneficial dose-response relationship between altitude and CVDs using a variety of data sources, including the National Center for Health Statistics, the National Elevation Dataset, and the U.S. Census Estimates. In addition, Winkelmayer *et al*. [65] reported a lower mortality rate for myocardial infarction, stroke, and CVDs among dialysis patients in people living at high altitudes.

Previous studies have evaluated the efficacy of hypoxic exposure as a potential therapeutic modality for increasing cardiovascular benefits and treating or preventing CVDs based on the beneficial relationship between altitude and CVDs [66, 67, 68, 69, 70, 71]. Vedam *et al*. [66] examined the effects of hypoxia corresponding to 80% $S_{\text{a}}$$O_{2}$ for 20 min and the accompanying changes in HR and BP on two components of arterial stiffness in healthy men. They reported that hypoxic exposure activates endothelium-derived nitric oxide (NO)-mediated mechanisms to induce acute vasodilation of arteries and increase blood flow in the skeletal muscle vascular bed despite enhanced sympathomimetic vasoconstriction. Leuenberger *et al*. [67] measured NO in forearm venous blood and skeletal muscle interstitial dialysate in seven healthy young men during 20 min of exposure to hypobaric hypoxia (simulated altitude 2438 m and 4877 m). They demonstrated that hypoxia plays an important role in the NO response in peripheral blood vessels as hypobaric hypoxia is associated with increased NO in venous effluent from skeletal muscle but not in the skeletal muscle interstitium. The hypoxia-induced skeletal muscle vasodilation response appears to be due to an increase in the release of vasodilators that play a central role in the NO/NO synthase pathway [17, 18, 23, 25]. Tremblay *et al*. [68] investigated the effects of blood viscosity, shear stress, and arterial endothelial function and the impact of plasma volume expansion during exposure to hypobaric hypoxia based on a crossover design in 11 healthy men. They confirmed that arterial endothelial function is maintained under hypobaric hypoxia and that plasma volume expansion improves arterial endothelial function in a shear-stress stimulus-specific manner.

Additionally, Lyamina *et al*. [69] examined the effect of a 20-day, intermittent, normobaric, intermittent hypoxia conditioning program on BP and NO production in patients with stage 1 arterial hypertension. They reported that the intermittent hypoxia conditioning program increased NO synthesis and decreased BP in hypertensive patients, and the reduction in BP persisted for at least 3 months in 28 of 33 hypertensive patients. Burtscher *et al*. [70] investigated the effectiveness of intermittent hypoxic exposure (IHE) (inspired oxygen fraction; $F_{\text{i}}$$O_{2}$ = 0.10–0.14) for 3 weeks by randomly assigning 16 men (50–70 years of age; 8 with myocardial infarction and 8 without prior myocardial infarction) in a double-blind manner. They concluded that IHE for 3 weeks showed an improvement in aerobic capacity and exercise tolerance in elderly men with and without CAD. The effect of intermittent hypobaric hypoxia on myocardial perfusion in patients with coronary heart disease was investigated by del Pilar Valle *et al*. [71]. They reported that hypoxic exposure improved myocardial perfusion in patients with severe coronary heart disease and that intermittent hypobaric hypoxia could be an alternative for the management of patients with chronic coronary heart disease.

Based on the previous studies discussed above, hypoxic exposure enhances arterial endothelial function through activation of NO-mediated mechanisms to increase cardiovascular benefit and shows promise as a potential therapeutic modality for treating or preventing CVDs.

### 3.2 Exercise Intervention Under Hypoxia as a Therapeutic Modality for Cardiovascular Benefit

The addition of physical activity to hypoxia results in compensatory vasodilatation where there is increased blood flow to the activated skeletal muscles, compensating for reduced oxygen content in arteries and keeping oxygen delivery to active muscles relatively constant [72]. Acute exercise and/or exercise interventions are independent and highly potent metabolic stressors [73]. Acute hypoxic exposure reduces $S_{\text{a}}$$O_{2}$, but exercise increases maximal oxygen uptake by working skeletal muscles [74]. Acute exercise and exercise intervention under hypoxia considerably reduces the oxygen partial pressure within the mitochondria of the working skeletal muscles by simultaneously decreasing oxygen supply and increasing oxygen demand [74]. This results in increased production of NO, a major contributor to the compensatory vasodilator response, in vascular endothelial cells in an oxygen-sensitive manner. These physiological responses result in increased production of NO, a major contributor to the compensatory vasodilator response, in vascular endothelial cells in an oxygen-sensitive manner [25, 73, 74]. This compensatory vasodilatation means that exercise interventions under hypoxia can effectively enhance cardiovascular benefits and act as a modality to prevent and treat CVDs.

Regarding acute exercise under hypoxia, Jung *et al*. [36] investigated the effect of an acute pilates program under hypoxia on metabolic, cardiac, and vascular functions in healthy women. They concluded that, compared to normoxic conditions, the acute pilates program under hypoxia ($F_{\text{i}}$$O_{2}$ = 0.145) led to greater metabolic and cardiac responses and elicited an additive effect on vascular endothelial function. Katayama *et al*. [75] investigated the effect of acute exercise under hypoxia ($F_{\text{i}}$$O_{2}$ = 0.12) on flow-mediated vasodilation (FMD) in eight healthy men. They suggested that acute exercise under hypoxia has a significant impact on endothelial-mediated vasodilation compared to normoxia. Although these findings were performed in a healthy population, they may provide evidence that exercise under hypoxia improves cardiovascular benefits.

Exercise intervention under hypoxic conditions has been studied and has shown a clear therapeutic effect on cardiovascular health. Nishiwaki *et al*. [33] examined the effects of exercise training under hypobaric hypoxia (600.1–608.3 mmHg; simulated 2000 m altitude) on arterial stiffness and FMD in 16 postmenopausal women (56 ± 1 years). They reported that exercise intervention under hypoxia induces improved vascular health and an increase in FMD, and these findings may have important implications for the development of new and effective exercise regimen programs. Park *et al*. [76] examined the effect of exercise intervention under hypoxia ($F_{\text{i}}$$O_{2}$ = 0.145) for the ANS in older men and reported that hypoxic training, compared with normoxic training, is a novel and successful ANS promotion modality in older men. Jung *et al*. [37] examined the effect of pilates training under hypoxia ($F_{\text{i}}$$O_{2}$ = 0.145) on cardiovascular risk factors, arterial stiffness, FMD, and hemorheological properties in obese women. They reported that hypoxic pilates intervention elicited a decrease in BP and cardiovascular risk factors and an increase in FMD, erythrocyte deformability, and erythrocyte aggregation in obese women compared with pilates intervention under normoxia. Zembron-Lacny *et al*. [77] investigated the effects of 6-day intense physical activity with IHE on oxi-inflammatory mediators and their interaction with conventional CVD risk factors. They demonstrated that IHE ($F_{\text{i}}$$O_{2}$ = 0.135 and $F_{\text{i}}$$O_{2}$ = 0.12) combined with sports activity reduced the risk of endothelial dysfunction and atherogenesis in athletes even though the oxi-inflammatory processes were enhanced. These results suggest that exercise intervention under hypoxia is a potential therapeutic and non-pharmacological method for reducing CVD risk in elite athletes participating in vigorous training. Additionally, Wee and Climstein [30] evaluated the effectiveness of exercise intervention under hypoxia on the modulation of cardiometabolic risk factors via a systemic review. They concluded that exercise intervention under hypoxia may be used as an adjunct therapeutic modality to modify some cardiometabolic risk factors such as body composition, glucose tolerance, lipid profiles, and BP.

In previous studies conducted with patients with CVDs, Korkushko *et al*. [78] investigated the efficacy of exercise training under hypoxia in the elderly with CAD, and they confirmed that hypoxic training is a non-pharmacological therapy that can effectively improve CAD through the economic function of the cardiovascular system, optimization of oxygen consumption, improvement of vasomotor endothelial function due to increased NO formation, and normalization of microcirculation. Serevrovskaya and Xi [24] conducted a review study on the practical effectiveness of exercise intervention under hypoxia as a non-pharmacological treatment for CVDs by synthesizing several studies. Based on evidence accumulating from studies of the last 50 years in healthy populations and patients with CVDs, hypoxic therapy modality, composed of 3–4 bouts of 5–7 min exposures to 12–10% oxygen alternating with normoxic durations for 2–3 weeks, can result in remarkable beneficial effects on cardiovascular health and the treatment of CVDs such as hypertension, coronary heart disease, and heart failure. Muangritdech *et al*. [32] examined the effects of 6-week exercise interventions under hypoxia ($F_{\text{i}}$$O_{2}$ = 0.14) on BP, NO metabolites (NOx), and hypoxia-inducing factor-1 alpha levels (HIF-1α) in 47 hypertensive patients. They concluded that exercise intervention under hypoxia may act as an alternative therapeutic strategy for hypertension patients, probably through the elevation of NOx and HIF-1α production.

In summary, hypoxic therapy, such as acute exercise and/or exercise intervention under hypoxia, induces greater reductions in BP and improves vascular endothelial function and hemorheological properties, thereby reducing cardiovascular risk and improving various aspects of cardiovascular function compared to circumstances under normoxia [24, 33, 36, 37, 62, 63, 64, 65, 66, 67, 68, 69, 70, 71, 75, 77, 78, 79]. This evidence confirms that hypoxic therapy is a promising modality for improving cardiovascular benefits and in preventing and treating CVDs.

## 4. Precautions of Hypoxic Therapy on Cardiovascular Disease

Hypoxic therapy is a novel therapeutic modality that may improve cardiovascular benefit and be utilized in the prevention and treatment of CVDs, but further clinical trials and thorough evaluation are needed to standardize various hypoxic therapies and utilize hypoxic devices [24]. Levine [80] reported that there are several considerations associated with taking patients with CVDs to hypoxia as follows: (1) the decrease in available oxygen in hypoxia can cause or worsen the symptoms of CVDs; (2) hypoxia and various environmental conditions (exercise, dehydration, changes in diet, thermal stress, and emotional stress from personal danger or conflict) can cause acute CVDs; (3) occasionally, sudden death from CVDs may occur; (4) ensuring optimal health, allowing adequate compliance for at least 5 days, and optimizing the intake of medications such as statins and aspirin are important in reducing the risk of side effects; (5) evaluation of exercise capacity and ischemia through a graded exercise test in normoxia is needed, and the decision of a specialist is warranted for participation in hypoxic therapy. These considerations mean that first aid and medical systems must be thoroughly equipped when hypoxic therapy is used for patients with CVDs.

## 5. Conclusions

Hypoxic therapy, including exposure or exercise interventions under hypoxic conditions, can be utilized as a novel therapeutic modality for cardiovascular benefit and improvement of CVDs based on a variety of physiologic and pathologic responses. In particular, with regard to cardiovascular health and CVDs, many previous studies have reported that various hypoxic therapies have positive effects on BP, cardiovascular risk factors, arterial stiffness, FMD, hemorheological properties, NO, NOx, and HIF-1α in healthy populations and CVD patients. This evidence confirms that hypoxic therapy is a promising modality for improving cardiovascular benefits and in preventing and treating CVDs. However, it is considered that hypoxic therapy can be stably applied to the prevention and treatment of CVDs only after various clinical trials and thorough evaluation to standardize various hypoxic therapies and to apply appropriate hypoxic devices.

## Abbreviations

CVDs, cardiovascular diseases; CAD, coronary artery disease; GLP-1, glucagon-like peptide-1; PP, pancreatic polypeptide; PYY, peptide YY; $S_{\text{a}}$$O_{2}$, arterial oxygen saturation; BP, blood pressure; NO, nitric oxide; FMD, flow-mediated vasodilation; IHE, intermittent hypoxic exposure; ANS, autonomic nervous system; NOx, nitric oxide metabolites; HIF-1α, hypoxia-inducible factor-1 alpha levels.
